# Patterns of recurrence after selective postoperative radiation therapy for patients with head and neck squamous cell carcinoma

**DOI:** 10.1186/s12885-016-2229-x

**Published:** 2016-03-07

**Authors:** Naoya Murakami, Fumihiko Matsumoto, Seiichi Yoshimoto, Yoshinori Ito, Taisuke Mori, Takao Ueno, Keisuke Tuchida, Tairo Kashihara, Kazuma Kobayashi, Ken Harada, Mayuka Kitaguchi, Shuhei Sekii, Rei Umezawa, Kana Takahashi, Koji Inaba, Hiroshi Igaki, Jun Itami

**Affiliations:** Department of Radiation Oncology, National Cancer Center Hospital, 5-1-1 Tsukiji, Chuo-ku, Tokyo, 104-0045 Japan; Department of Head and Neck Surgery, National Cancer Center Hospital, 5-1-1 Tsukiji, Chuo-ku, Tokyo, 104-0045 Japan; Department of Clinical Laboratory and Pathology, National Cancer Center Hospital, 5-1-1 Tsukiji, Chuo-ku, Tokyo, 104-0045 Japan; Department of Oral Health and Diagnostic Sciences, National Cancer Center Hospital, 5-1-1 Tsukiji, Chuo-ku, Tokyo, 104-0045 Japan

**Keywords:** Head and neck squamous cell carcinoma, Postoperative radiation therapy, Patterns of recurrence, Selective neck irradiation

## Abstract

**Background:**

The radiation field for patients with postoperative head and neck squamous cell carcinoma is narrower in our institution than in Western countries to reduce late radiation related toxicities. This strategy is at a risk of loco-regional or distant metastasis. However, because patients are more closely checked than in Western countries by every 1 to 2 months intervals and it is supposed that regional recurrences are identified and salvage surgeries are performed more quickly. Therefore, it is considered that patient survival would not be compromised with this strategy. The aim of this study was to investigate the feasibility of this strategy retrospectively.

**Methods:**

Patients who underwent neck dissection with close or positive margin, extra-capsular spread (ECS), multiple regional lymph node metastasis, pT4, with or without primary tumor resection were treated with postoperative radiation therapy. The volume of radiation field, especially the coverage of prophylactic regional lymph node area, was discussed among head and neck surgeons and radiation oncologists taking into account the clinical factors including patient’s age, performance status, number of positive lymph nodes, size of metastatic lymph nodes, extension of primary tumor beyond the midline, and existence of ECS.

**Results:**

Seventy-two patients were identified who were treated with postoperative radiation therapy for head and neck squamous cell carcinoma between November 2005 and December 2014. There were 20 patients with oropharynx, 19 with hypopharynx, 7 with larynx, 23 with oral cavity, and 3 with other sites. Thirty eight patients had their neck irradiated bilaterally and 34 unilaterally. Median follow-up period for patients without relapse was 20.7 months (5.1–100.7). Thirty two patients had disease relapse after treatment including 22 loco-regional recurrence and 14 distant metastases. Among 22 loco-regional recurrence, seven patients underwent salvage surgery and one of them was no relapse at the time of the analysis. Among patients without bilateral neck lymph node metastasis who were treated with unilateral neck irradiation, patients with oral cavity or recurrent disease had significantly lower DFS compared with those without (2-y DFS 41.7 % vs 88.2 %, *p* = 0.017).

**Conclusions:**

In patients without bilateral neck lymph node involvement, the postoperative unilateral neck irradiation is a reasonable treatment strategy for patients with the exception of oral cavity or recurrent disease.

## Background

According to statistics from Cancer Information Service in Japan, death from head and neck malignant tumors in Japan (malignant tumors arising from oral cavity, pharynx, and larynx) was 8142 in 2013 and this figure accounts for 2.2 % of all the death from malignant tumors [1]. Although the percentage is decreasing, the smoking rate in 2013 was 32.2 % in male and 8.2 % in female and still many people smoke in our country [2].

In 1970’s, Radiation Therapy Oncology Group (RTOG) 73–03 trial was carried out to compare preoperative with postoperative radiation therapy combined with surgical resection for patients with advanced operable squamous cell carcinoma of the supraglottic larynx or hypopharynx in the context of a phase III study [3]. Loco-regional control was significantly favorable for patients assigned to postoperative radiation therapy compared with those assigned to preoperative radiation therapy (65 % vs 48 %, *p* = 0.04), and the postoperative radiation therapy has been a standard of care for patients with advanced resectable head and neck squamous cell carcinoma (HNSCC). Nevertheless, development of distant metastasis was frequently observed in both arms and the addition of chemotherapy to surgery and adjuvant radiation therapy was considered as a next important issue. In the Intergroup study 0034 (or RTOG 85–03), a randomized clinical trial was conducted by cooperative groups which was consisted of RTOG, Southwest Oncology Group (SWOG), Eastern Cooperative Oncology Group (ECOG), Cancer and Leukemia Group B (CALGB), Northern California Oncology Group (NCOG), and Southwest Group (SEG), patients with advanced HNSCC were randomly assigned either to postoperative radiation alone or sequential three cycles of cis-platinum and 5-FU followed by postoperative radiotherapy [4]. While distant metastasis-free survival was significantly improved in sequential CT/RT arm (23 % vs 15 %, *p* = 0.02), both loco-regional relapse-free survival and overall survival did not differ between the two arms and the concomitant use of chemotherapy and radiation therapy was awaited. In 2004, the European Organization for Research and Treatment of Cancer (EORTC) and RTOG published simultaneously the results of two phase III trials (the EORTC 22931 and the RTOG 95–01) which compared concurrent postoperative chemoradiation using tri-weekly 100 mg/m^2^ of cis-platinum with postoperative radiotherapy alone [5, 6]. There were slight differences in settings between these two phase III clinical trials. In the RTOG 95–01, the primary endpoint was the rates of local and regional control whereas, in the EORCT 22931 it was chosen to be the progression-free survival. The definition of the high-risk characteristics also differed between these two trials. In the RTOG 95–01, the following characteristics were defined as high-risk; histologic evidence of invasion of two or more regional lymph nodes, extra-capsular spread (ECS) of nodal disease, and microscopically involved mucosal margins of resection. On the other hand, in the EORTC 22931, the following characteristics were defined as high-risk; ECS, positive resection margins, perineural involvement, vascular tumor embolism, or tumors with involved lymph nodes at level IV or V from carcinomas arising in the oral cavity or oropharynx. While primary endpoint of these two phase III clinical trials were both met and overall survival benefit was demonstrated in the EORTC 22931 trial (*p* = 0.02), the RTOG 95–01 showed only a trend in the same direction in overall survival (*p* = 0.19). Bernier et al. conducted a comparative analysis using data pooled from the EORTC 22931 and the RTOG 95–01 to identify which patients require adjuvant concomitant chemoradiation following surgery and they concluded that microscopically involved resection margins and ECS of tumor from neck nodes were the most significant adverse factors for poor outcome [7]. Therefore, concurrent chemoradiation (cCRT) is a standard therapy for postoperative high-risk HNSCC patients.

Originally the radical neck dissection (RND) consists of removal of all the lymphatic as well as non-lymphatic structures from the mastoid process down to the clavicle except the carotid artery, brachial plexus, hypoglossal, lingual, vagus, and phrenic nerves [8, 9], which demands heavy burden to patients. Later on, selective neck dissection (SND) was introduced which preserved one or more lymph node levels [10] and the development of common terminology of discriminating neck levels which was well-known as the classification of American Head and Neck Society (AHNS) followed [11, 12]. However, the applicability of the concept of the selective nodal irradiation in postoperative setting is controversial [13–15]. Gregoire et al. [13] and Chao et al. [14] proposed the clinical target volume (CTV) guidelines for postoperative neck region, but the authors admitted the paucity of data on which one could create a specific guideline for postoperative CTV. According to the guideline of Chao et al., only patients with buccal T1-2 N0 and tonsil T1-2 N0 were allowed for hemi-neck postoperative radiation. After extensive neck irradiation patients usually are suffered from late radiation toxicities, in especially chemotherapy was administered concurrently with radiotherapy. In our institution radiation field for patients with postoperative head and neck squamous cell carcinoma is narrower than in Western countries to reduce late radiation related toxicities. This strategy is at a risk of loco-regional recurrence and/or distant metastasis. However, because patients are checked closely by every 1 to 2 months intervals and the salvage surgery would be performed immediately after the identification of the regional recurrences, therefore, it is considered that the patient’s survival would not be compromised with this treatment strategy. This retrospective study was conducted to investigate the feasibility of this strategy.

## Methods

All consecutive patients with HNSCC who underwent neck dissection and received postoperative radiation therapy were recruited for this study. In our institution, patients with HNSCC who underwent neck dissection with pathologic findings of close or positive resection margin, ECS, multiple regional lymph node metastasis, or pT4, with or without primary tumor resection were treated with postoperative radiation therapy. Surgical margin status was defined as follows; close margins were defined as ≤ 3 mm and positive margins defined as tumor touching an inked surface.

From April 2011 concurrent chemoradiation (cCRT) with tri-weekly CDDP 80 mg/m^2^, and from March 2013 Cetuximab-radiation according to the Bonner protocol [16] was introduced in our institution for patients with positive resection margin or ECS. Because there is no evidence supporting the superiority of Cetuximab-radiation over platinum-based cCRT in the management of advanced HNSCC, our first choice was cCRT. However, if patients did not have enough kidney function with favorable performance status, Cetuximab-radiation was chosen.

From June 2009 neoadjuvant chemotherapy (NAC) was started as chemoselection for patients with advanced HNSCC who required total laryngectomy or who expected severe postoperative pharyngeal dysfunction. If favorable response was achieved after two to three cycles of induction chemotherapy, subsequent cCRT was followed with or without neck dissection. If not, total laryngectomy or appropriate surgery was applied. NAC was also applied as induction chemotherapy for patients with far-advanced disease for whom it was impossible to separate metastatic lymph nodes from carotid artery which precludes radical operation or patients with N2c and/or lower neck metastasis who’s possibility of developing distant metastasis soon after surgery was expected to be very high. Agents used for NAC was either the combination of CDDP and 5FU or CDDP, 5FU, and docetaxel.

The extent of prophylactic neck resection was determined by the status of primary lesion. If primary lesion extended midline, prophylactic contralateral neck dissection was applied. Otherwise, unilateral prophylactic neck dissection was performed.

Patients with distant metastasis, treated for palliative intention, or for salvage intention after recurrence without surgical resection were excluded from this study.

### Radiotherapy

Radiotherapy was prescribed in 2-Gy fractions with 4 or 6-MV photons in either three-dimensional conformal radiotherapy (3DCRT) or intensity-modulated radiotherapy (IMRT). From September 2008 a simultaneous integrated boost intensity-modulated radiotherapy (SIB-IMRT) using sliding window technique or volumetric modulated arc therapy (VMAT) by dynamic MLC system (Varian Medical Systems, Palo Alto, CA) was introduced in our institution, in case of the CTV contained large volume of major salivary gland, oral cavity, larynx, or pharynx. Our IMRT procedure for head and neck cancer patients is described in a previous report [17]. Patients were immobilized from head to shoulders with thermoplastic masks in the supine position. Target volumes were defined as follows: no gross tumor volume (GTV) was defined except patients without primary lesion resection because all gross tumor was resected during operation. The high-risk CTV 60–66 Gy (CTV_60-66Gy_) was defined as areas considered as high risk for having microscopic disease such as positive surgical margin or metastatic lymph node with ECS based on preclinical imaging, preoperative physical exam/endoscopy, operative findings, and final pathologic findings. The intermediate-risk CTV (CTV_44Gy_ for 3DCRT and CTV_54Gy_ for IMRT) included the cervical lymphatic pathways which are considered to be at risk for having potential microscopic disease. The extent of the CTV_44Gy_ or the CTV_54Gy_ was discussed among head and neck surgeons and radiation oncologists taking into account of clinical factors including patient’s age, performance status, number and distribution of positive lymph nodes, size of metastatic lymph nodes, extension of primary tumor beyond the midline, pathological resection status, and existence of ECS. For example, patients with ipsilateral multiple neck lymph node metastases with large nodes more than 3 cm in diameter and/or multiple ECSs generally received prophylactic contralateral neck irradiation. However, if patients were elderly and fragile, prophylactic contralateral neck irradiation was often omitted. If patients received total laryngectomy, the risk of acquiring aspiration pneumonia was reduced, therefore, threshold of providing prophylactic contralateral neck irradiation would be lowered.

After completion of radiotherapy, patients were closely followed by every 1 to 2 months for the initial 2 years, every 3 to 4 months for years 3–5, and once or twice a year thereafter. When surgically resectable recurrent lymph nodes were identified in the regional neck area without distant metastasis during the follow-up visits and patients had favorable performance status, a salvage surgery would be performed immediately after the identification.

### Statistics

Survival curves were estimated by using the Kaplan-Meier method and the differences were assessed by the log-rank test. The relationships between clinical and treatment variables and disease-free survival (DFS) were analyzed by the univariate analysis. Student’s unpaired *t* test was used to compare the continuous variables and Pearson’s chi-square test to compare the categorical variables. A P value of ≤0.05 was considered statistically significant. Factors with *p* value ≤0.05 were further analyzed in the multivariate analysis by the Cox regression analysis. This analysis was intended to find out a most appropriate population suitable for postoperative unilateral-RT, so the promising factors were combined and analyzed by the multivariate analysis. However, to eliminate the statistics confounding, factors were used in the multivariate analysis only once. The Statistical analysis was performed using SPSS Statistics (version 18.0; SPSS, Inc., Chicago, IL).

This retrospective study was approved by the institutional ethical review board of the National Cancer Center Hospital. This retrospective study was performed in accordance with the ethical standards laid down in the 1964 Declaration of Helsinki and its later amendments.

## Results

From November 2005 and December 2014, 72 patients were identified who underwent neck dissection and postoperative radiation therapy for HNSCC. Pretreatment patient and tumor characteristics are summarized in Table 1. There were 20 patients with oropharynx, 19 with hypopharynx, 7 with larynx, 23 with oral cavity, and 3 with other sites. Because HPV infectious status has been routinely assessed since 2011, only 6 out of 20 patients of oropharyngeal cancer patients were assessed for HPV and 5 of them (83.3 %) were positive for HPV. Two patient were stage III, 48 IVA, 3 IVB, and 19 after salvage surgery for recurrent disease. In recurrent cases, they were classified into either stage rIII or rIVA. Thirty eight patients had their neck irradiated bilaterally and 34 unilaterally. There was no difference between bilateral and unilateral neck irradiated cohorts except number of lymph node metastases. Statistically more patients had more than two lymph node metastases in bilateral neck cohort than unilateral neck cohort (*p* = 0.031), suggesting that more advanced patients were treated by bilateral neck irradiation. There was one patient with N2c who received unilateral neck irradiation. Because this 71 years old patient had past history of subtotal esophagectomy, left upper lobe segmentectomy, and major depression, and his contralateral side of neck lymph node was without ECS, therefore, it was decided that contralateral neck should be omitted for prophylactic irradiation to reduce toxicity. Pathological characteristics and treatment details are summarized in Table 2. Eight patients received NAC before surgery. Seven patients received the combination of CDDP and 5FU and one the combination of docetaxcel, CDDP, and 5FU. Three patients had their primary lesion treated by brachytherapy and nine patients by external beam radiation therapy. The others had their primary lesion as well as regional neck area surgically resected. Among 60 patients whose primary tumor had surgically resected, 38 patients had their primary site irradiated mainly because of positive/close resection margin or T4 disease. Statistically fewer patients were treated by 3DCRT in unilateral neck cohort than in bilateral neck cohort (*p* = 0.031).

**Table 1:** Patient and tumor characteristics

	Bilateral RT	Unilateral RT	
	(*n* = 38)	(*n* = 34)	*p value*
Primary site			
Oral cavity	10	13	0.556
Oropharynx	12	8	
Hypopharynx	10	9	
Larynx	5	2	
Others	1	2	
T-classification			
T1	4	4	0.673
T2	13	11	
T3	5	4	
T4	8	4	
Rec	8	11	
rT0	6	8	
rT1	0	0	
rT2	2	2	
rT3	0	0	
rT4	0	1	
N-classification			
N0	0	2	0.124
N1	0	0	
N2a	3	2	
N2b	18	17	
N2c	8	1	
N3	1	1	
Rec	8	11	
rN1	1	1	
rN2	7	10	
rN3	0	0	
Stage			
III	1	1	0.637
IVA	28	20	
IVB	1	2	
Rec	8	11	
rIII	1	1	
rIVA	7	10	
Bilateral neck LN metastasis			
Yes	9	1	0.011^*^
No	29	33	
Necrosis in LN			
Yes	21	21	0.401
No	14	9	
Unknown	3	4	
Maximum diameter of LN (cm)			
Median	2.5	2.7	0.783
Range	0.4–7.5	0.8–4.1	
Number of LN metastasis			
0–1	6	13	0.031^*^
≥ 2	32	21	
Sex			
Male	32	26	0.217
Female	6	8	
Age			
Median	63	63	0.270
Range	38–80	34–84	

*RT* radiation therapy, *Rec* recurrence, *LN* lymph node. *A *P* value of ≤0.05 was considered statistically significant

**Table 2:** Pathological characteristics and treatment details

NAC	
Yes	8
No	64
Bi-lateral ND	
Yes	26
No	46
Treatment for primary lesion	
Surgery	60
EBRT	9
BT	3
Degree of differentiation	
Poorly differentiated	20
Moderately differentiated	23
Well differentiated	15
Unknown	14
Extracapsular spread	
Yes	46
No	10
Unknown	16
Positive/close margin	
Yes	40
No	32
Concurrent systemic therapy	
CDDP	17
Cetuximab	2
TS-1	2
None	51
RT total dose (Gy)	
Median	66
Range	60–74
Radiation technique	
3DCRT	14
IMRT	58

*RT* radiation therapy, *NAC* neoadjuvant chemotherapy, *ND* neck dissection, *EBRT* external beam radiation therapy

*BT* brachytherapy, *LN* lymph node, *3DCRT* three-dimensional conformal radiation therapy, *IMRT* intensity modulated radiation therapy

**Fig. 1:**
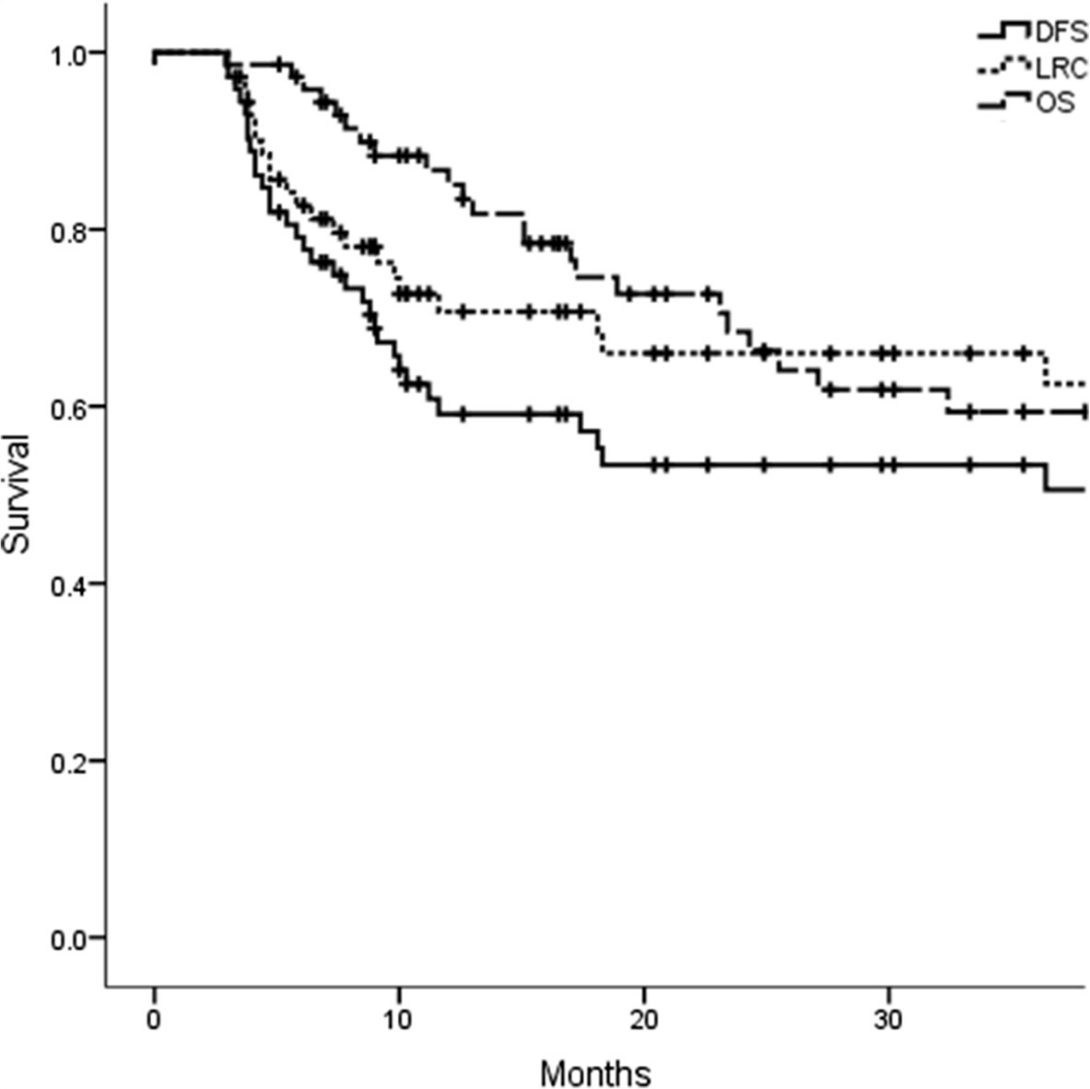
Kaplan-Meyer curves of overall survival (OS), disease-free survival (DFS), and loco-regional control (LRC)

**Fig. 2:**
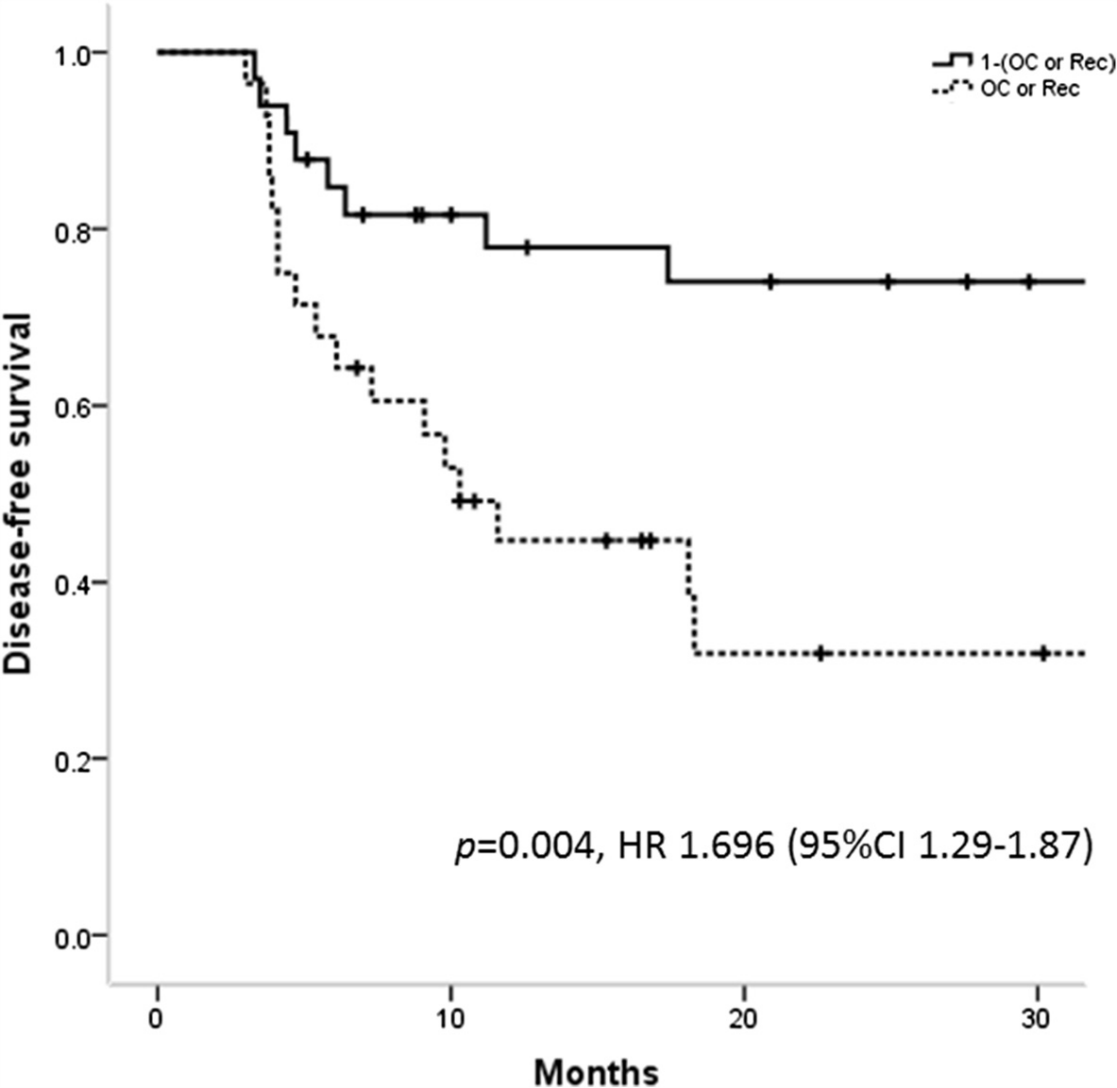
Disease-free survival (DFS) stratified by the group of patients with oral cavity or recurrent disease or those without

**Fig. 3:**
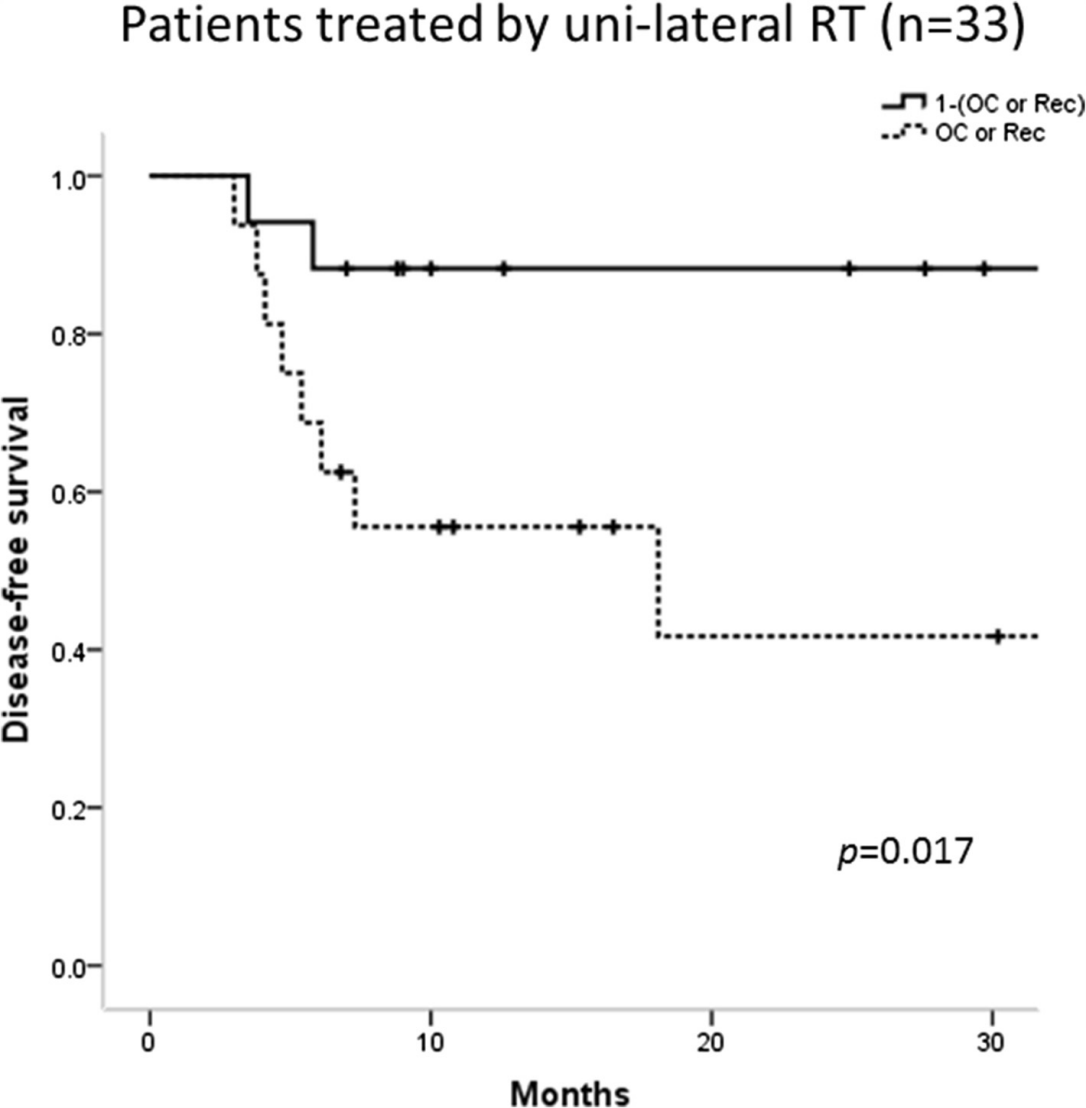
Disease-free survival (DFS) for patients treated by unilateral neck cohort. Survival curves were stratified by the group of patients with oral cavity or recurrent disease or those without

Median follow-up period for patients without failure was 20.7 months (range, 5.1–100.7 months). 2-year Overall survival (OS), DFS, and Loco-regional control (LRC) were 66.0, 53.4, and 66.0 %, respectively (Fig. 1). Pattern of the first relapse is summarized in Table 3. Thirty two patients had disease relapse after treatment including 22 loco-regional recurrence and 14 distant metastases. Significantly more patients with failure were identified in bilateral neck cohort (*p* = 0.015). Ten patients developed in-field recurrence. Nodal failure was found within the high-risk CTV in seven patients and within the intermediate-risk CTV in three patients. Twelve patients were identified with extra-field loco-regional failure: three recurrences were found in ipsilateral retropharyngeal lymph node, one in ipsilateral level Ib and IV simultaneously, two in ipsilateral level V, two in tumor bed region, one in the nasopharynx, two in neck skin, and one in non-irradiated contralateral neck region. The most frequently affected site as a distant metastasis was lung, following bone and mediastinal lymph node. Among 22 loco-regional recurrence, seven patients underwent salvage surgery although only one patient remains without relapse at the time of the analysis. Potential factors influencing DFS were summarized in Table 4. In the univariate analysis, IMRT was found out to be factors for favorable DFS. On the other hand, more than two lymph node metastasis, oral cavity primary or recurrent disease, T4 or recurrent disease, and oral cavity primary or recurrent disease were identified to be factors for unfavorable DFS. The aim of this study was to find a group of patients who could be safely applied unilateral neck irradiation and generally it is natural to irradiate bilateral neck for patients with bilateral neck lymph node involvement. Consequently, all 10 patients with bilateral neck lymph node metastasis were excluded and uni- and multivariate analysis was performed (Table 5). In the multivariate analysis, it was found that inferior DFS correlated with oral cavity or recurrent disease (Hazard Ratio 1.696; 95 % confidence interval 1.29–1.87, Fig. 2). Among patients with unilateral lymph node metastasis treated with unilateral neck irradiation, oral cavity or recurrent disease were adverse factors for DFS (2-y DFS 41.7 % vs 88.2 %, *p* = 0.017, Fig. 3). On the other hand, among patients who had unilateral lymph node metastasis treated with bilateral neck irradiation, no statistically significant difference was found but a tendency towards inferior DFS for patients with oral cavity or recurrent disease compared to that of those without (2-y DFS 22.2 % vs 60.9 %, *p* = 0.056). Out of 40 relapse-free patients, one patient was para-enteral nutrition dependent, two patients developed hypothyroidism requiring the hormone replacement treatment, one patient developed ulcer at tonsil which resolved conservatively, one patient developed severe dry mouth which always required water to moisten the mouth, two patients developed metachronous malignancy in head and neck region, and two patients died of intercurrent disease (one died of subarachnoid hemorrhage and one liver cirrhosis). The one who remained para-enteral nutrition dependent was treated by bilateral neck irradiation.

**Table 3:** Pattern of first failures

	Bilateral RT	Unilateral RT	
	(*n* = 38)	(*n* = 34)	*p value*
Any failure			
Yes	22	10	0.015*
No	16	24	
Loco-regional failure			
Yes	15	7	0.082
No	23	27	
In-field failure			
Yes	8	2	0.062
No	30	32	
Extra-field loco-regional failure			
Yes	7	5	0.572
No	31	29	
Distant failure			
Yes	10	4	0.119
No	28	30	

*RT* radiation therapy. *​A *P* value of ≤0.05 was considered statistically significant

**Table 4:** Potential predictors influencing DFS

	2-y DFS (%)		DFS
	yes	no	*p value*
OC	38.9	59.8	0.067
Necrosis in LN	63.8	49.1	0.245
Bi-lateral ND	50.3	54.7	0.654
NAC	87.5	48.6	0.076
Rec	30.6	59.6	0.062
T4	37.5	56.2	0.21
LN+ ≥2LN+ ≥2	44.9	76.5	0.019*
Extracapsular extention	50.7	62.5	0.177
Positive/close margin	48.5	57.2	0.82
Bilateral RT	42	68.4	0.057
IMRT	69.5	35.7	0.047*
Systemic therapy	38.7	57.1	0.449
OC or rec	33.9	66.4	0.013*
T4 or rec	36.7	65.7	0.012*
OC or T4	37.5	65.1	0.012*
OC or T4 or rec	37.9	72.4	0.006*

*DFS* disease free survival

*OC* oral cavity, *LN* lymph node, *ND* neck dissection, *Rec* recurrence. *​A *P* value of ≤0.05 was considered statistically significant

**Table 5:** Potential predictors influencing DFS for patients excluding bilateral neck lymph node metastasis

	2-y DFS (%)		DFS			
	yes	no	*p* value in uni.	*p* value in multi.	HR	95 % CI
OC	36.9	64.4	0.034*			
Necrosis in LN	64.9	48.4	0.423			
Bi-lateral ND	53.0	55.9	0.998			
NAC	87.5	48.6	0.076			
Rec	30.6	64.2	0.029*			
T4	46.7	56.6	0.398			
LN+ ≥2LN+ ≥2	45.3	76.5	0.032*	0.062		
Extracapsular extention	52.2	57.1	0.442			
Positive/close margin	56.1	54.2	0.824			
Bilateral RT	44.4	67.3	0.146			
IMRT	60.8	33.3	0.050*	0.162		
Systemic therapy	43.3	56.5	0.619			
OC or rec	31.9	74.0	0.004*	0.006	1.696	1.29–1.87

*DFS* disease free survival, *uni.* univariate analysis, *multi*. multivariate analysis, *HR* hazard ration, *CI* confidence interval, *OC* oral cavity, *LN* lymph node, *ND* neck dissection, *Rec* recurrence. *​A *P* value of ≤0.05 was considered statistically significant

## Discussion

The relapse rate was significantly higher in bilateral neck cohort compared with unilateral neck cohort although larger volume being irradiated (Table 3, *p* = 0.015). The possible explanation of this unfavorable results in bilateral neck cohort was that statistically more patients with two or more lymph node metastases were treated by bilateral radiation therapy (Table 1).

Eisbruch et al. reported that there existed dose-volume relationship between the pharyngeal constrictors, the glottic, and supraglottic larynx and late radiation complications such as dysphagia and aspiration [18]. Because our study was only a retrospective study and it was not possible to collect reliable data concerning late neck toxicities. Therefore, it was not possible to show inferior quality of life for patients who were treated by bilateral neck irradiation compared with those who were treated by unilateral neck irradiation. However, it is common in daily practice to see patients with bilateral neck irradiation who are suffered from neck stiffness or shoulder discomfort which not merely worsen patient’s quality of life but also hinders early detection of neck lymph node recurrence or make it difficult to perform possible salvage surgery. Therefore, if it is feasible, to reduce acute and late complications related to irradiation it is obviously desirable to irradiate as smaller volume as possible. There were only two patients with N0 in this study (Table 1), but large T classification T3 with positive margin and T4. Hence, if the guideline for postoperative radiation therapy created by Chao et al. [14] would have been applied to our patients, theoretically all the patients should have been treated by bilateral neck irradiation. This patient with T3N0 and positive margin eventually developed local and regional recurrence. Because there were only two patients with postoperative N0 in our study, it is difficult to make any recommendations of postoperative radiation therapy for postoperative N0 patients. However, because one among the two N0 patients developed regional recurrence, prophylactic postoperative radiation seems to be also important for postoperative N0 patients with high risk pathological features.

It was observed in this study that seven out of 22 patients with loco-regional recurrence could undergo salvage surgery and only one of them eventually achieved no relapse at the time of the analysis. Therefore, salvage surgery had only minor impact on patient’s overall survival. This finding was in line with a recent randomized phase III trial comparing elective neck dissection or watchful wait with close follow up for early-stage oral cancer. The latter strategy was significantly inferior in overall survival rate despite the protocol mandated close follow-up for neck examination [19]. In this study, patients who developed nodal relapse presented with a more advanced nodal stage and a higher prevalence of ECS than initial presentation, which possibly made it more difficult to control disease by salvage interventions. Accordingly, finding patients who are unlikely to develop loco-regional recurrence after unilateral neck irradiation seems to be a better treatment strategy.

HNSCC with a positive human papilloma virus (HPV) has been recently reported to be radiosensitive [20, 21]. Ki-67 and p53 were also reported to be prognostic markers for HNSCC postoperative radiotherapy [22]. The prognostic impact of these markers on survival for patients with HNSCC who were treated with postoperative radiation therapy could not be assessed because only part of patients were examined for p16, Ki-67, and p53 status in this study. Similarly, although ECS of lymph node is a well-known major adverse pathological factor among patients of HNSCC [5–7] and description concerning ECS of lymph node has been found since 2005, it was only from 2011 that documentation about ECS of lymph node has been made without exception. Therefore, there were as many as 16 missing data and the prognostic impact of ECS of lymph node could not be found in our study. Resection margin status is also a well-known major adverse pathological factor [5–7]. However, in our study adverse prognostic feature of resection margin status could not be shown presumably because patients with positive/close margin received postoperative radiation therapy appropriately. On the other hand, multivariate analysis in this study revealed that patients with oral cavity or recurrent disease were significantly worse DFS compared with those without and its disease-free survival disadvantage was 69.6 % (Table 5, Fig. 2). Radiation resistance of tumors from oral cavity [23] has been reported previously, therefore, current findings were clinically comprehensible. Among patients without bilateral neck node metastasis and treated with unilateral neck irradiation, patients with oral cavity or recurrent disease had significantly inferior DFS compared with those without (2-y DFS 41.7 % vs 88.2 %, *p* = 0.017, Fig. 3). Therefore, in patients without bilateral neck lymph node involvement, the postoperative unilateral neck irradiation is a reasonable treatment strategy for patients with the exception of oral cavity or recurrent disease.

On the contrary, for patients with oral cavity origin or recurrent disease, bilateral neck irradiation did not seem to be a promising solution. If bilateral neck irradiation was a favorable solution for patients with oral cavity or recurrent disease, DFS should have been superior for patients with bilateral neck irradiation to those with unilateral neck irradiation. However, among patients with oral cavity or recurrent disease, 2-y DFS for patients with bilateral and unilateral neck irradiation were 22.2 % and 41.7 %, respectively (*p* = 0.412). Thus, different approaches should be made to improve the clinical results for patients with oral cavity or recurrent disease. In this study, the most frequent site of regional recurrence was the high-risk CTV (70 %), similar results showed with Carrillo et al. [15]. Out of field regional recurrence was observed more frequently in ipsilateral neck than contralateral neck whereas only one patient developed contralateral-neck failure. Because concurrent CDDP administration was only started since 2008 in our institution, the majority patients did not received cCRT in this analysis, which could be a possible explanation for many loco-regional recurrences. Therefore, dose escalation for the high-risk CTV or application of cCRT or widening the intermediate-risk CTV in ipsilateral neck region to submandibular or posterior neck would possibly decrease the rate of loco-regional recurrence in the future.

There were several limitations in this study. Treatment strategy and radiation field was not uniformed according to several patient’s clinical backgrounds. For example, treatment plans were heterogeneous including bioradiation, chemoradiation, or radiation alone. And the chemotherapy agents used were not unified. Also, this study was a retrospective study consisted of a small number of patients from single institution. In spite of these drawbacks, several insights were derived from this analysis which would possible improve treatment in the future.

## Conclusions

In patients without bilateral neck lymph node involvement, the postoperative unilateral neck irradiation is a reasonable treatment strategy for patients with the exception of oral cavity or recurrent disease.

## Abbreviations

3DCRT: three-dimensional conformal radiotherapy
AHNS: American Head and Neck Society
CALGB: Cancer and Leukemia Group B
cCRT: concurrent chemoradiation
CTV: clinical target volume
DFS: disease-free survival
ECOG: Eastern Cooperative Oncology Group
ECS: extra-capsular spread
EORTC: European Organization for Research and Treatment of Cancer
GTV: gross tumor volume
HNSCC: head and neck squamous cell carcinoma
HPV: human papilloma virus
IMRT: intensity-modulated radiotherapy
LRC: loco-regional control
NAC: neoadjuvant chemotherapy
NCOG: Northern California Oncology Group
OS: overall survival
RND: radical neck dissection
RTOG: Radiation Therapy Oncology Group
SEG: Southwest Group
SIB-IMRT: simultaneous integrated boost intensity-modulated radiotherapy
SND: selective neck dissection
SWOG: Southwest Oncology Group
VMAT: volumetric modulated arc therapy
